# The potential roles of gossypol as anticancer agent: advances and future directions

**DOI:** 10.1186/s13020-023-00869-8

**Published:** 2023-12-15

**Authors:** Danijela Paunovic, Jovana Rajkovic, Radmila Novakovic, Jelica Grujic-Milanovic, Reham Hassan Mekky, Dragos Popa, Daniela Calina, Javad Sharifi-Rad

**Affiliations:** 1 Institute for Biological Research Sinisa Stankovic, National Institute of the Republic of Serbia, University of Belgrade, Belgrade, Serbia; 2 Institute for Pharmacology, Clinical Pharmacology and Toxicology, Medical Faculty, University of Belgrade, Belgrade, Serbia; 3 Center for Genome Sequencing and Bioinformatics, Institute of Molecular Genetics and Genetic Engineering, University of Belgrade, 11042 Belgrade, Serbia; 4 Institute for Medical Research, National Institute of the Republic of Serbia, Department for Cardiovascular Research, University of Belgrade, Belgrade, Serbia; 5 Department of Pharmacognosy, Faculty of Pharmacy, Egyptian Russian University, Badr City, 11829 Cairo Egypt; 6 Department of Plastic Surgery, University of Medicine and Pharmacy of Craiova, 200349 Craiova, Romania; 7 Department of Clinical Pharmacy, University of Medicine and Pharmacy of Craiova, 200349 Craiova, Romania; 8 Facultad de Medicina, Universidad del Azuay, Cuenca, Ecuador

**Keywords:** Gossypol, Anticancer mechanisms, Apoptosis, Proliferation, Molecular targets, Mechanisms

## Abstract

Gossypol, a polyphenolic aldehyde derived from cottonseed plants, has seen a transformation in its pharmaceutical application from a male contraceptive to a candidate for cancer therapy. This shift is supported by its recognized antitumor properties, which have prompted its investigation in the treatment of various cancers and related inflammatory conditions. This review synthesizes the current understanding of gossypol as an anticancer agent, focusing on its pharmacological mechanisms, strategies to enhance its clinical efficacy, and the status of ongoing clinical evaluations.

The methodological approach to this review involved a systematic search across several scientific databases including the National Center for Biotechnology Information (NCBI), PubMed/MedLine, Google Scholar, Scopus, and TRIP. Studies were meticulously chosen to cover various aspects of gossypol, from its chemical structure and natural sources to its pharmacokinetics and confirmed anticancer efficacy. Specific MeSH terms and keywords related to gossypol’s antineoplastic applications guided the search strategy.

Results from selected pharmacological studies indicate that gossypol inhibits the Bcl-2 family of anti-apoptotic proteins, promoting apoptosis in tumor cells. Clinical trials, particularly phase I and II, reveal gossypol’s promise as an anticancer agent, demonstrating efficacy and manageable toxicity profiles. The review identifies the development of gossypol derivatives and novel carriers as avenues to enhance therapeutic outcomes and mitigate adverse effects.

Conclusively, gossypol represents a promising anticancer agent with considerable therapeutic potential. However, further research is needed to refine gossypol-based therapies, explore combination treatments, and verify their effectiveness across cancer types. The ongoing clinical trials continue to support its potential, suggesting a future where gossypol could play a significant role in cancer treatment protocols.

## Introduction

Gossypol is a polyphenol compound found in cotton plants (*Gossypium* sp.) It is a seed pigment with a protective role. It is also known as an oral male contraceptive for treating gynaecological disorders. Numerous studies have shown its anti-tumour, antioxidant, antiviral, antimicrobial, and immunomodulatory activities [28]. Nevertheless, gossypol has limited application in medicine as a potential pharmacological agent, mainly due to the narrow therapeutic range of doses, the risk of permanent irreversible sterility [72], and hypokalaemia. This problem led to numerous studies aimed at reducing the side effects and toxicity of gossypol and identifying and developing new derivative molecules with reduced side effects and toxicity. The mechanism of anticancer activity of gossypol is the induction of apoptosis through the suppression of anti-apoptotic proteins of the Bcl-2 family [85]. Anticancer activity of gossypol is proven on several different cancer cell lines [49]: human breast cancer cells (MCF-7, MDA-MB-231, MDA-MB-468, ZR-75-1, and T47D), pancreatic cancer cells (BxPC-3 and MIA PaCa-2), human colon cancer cells (COLO 225), human cervical cancer cells (HeLa and SiHa cell lines), non-small cell lung cancer (NSCLC) cell lines (H1975), human lung cancer cell lines (H1299 and H358) and prostate cancer cells. Except in China, where gossypol is available on the drug market as an adjuvant used for tumour treatment [85], in the rest of the world, gossypol is still under clinical trials investigation. This comprehensive study aims to summarise all available data on the biological properties of gossypol, particularly its anticancer activity, together with the mechanism of this activity and an overview of clinical studies with gossypol and its medical use.

## Review methodology

Information was gathered from various scientific databases, including the National Center for Biotechnology Information (NCBI), PubMed/MedLine, Google Scholar, Scopus and TRIP databases for this comprehensive review of gossypol and its potential anticancer activity. The selected studies were analysed about the structure and plant sources of gossypol and its derivatives, the medicinal use, the bioavailability and scientific studies that confirmed the anticancer properties of the compound. The following MeSH terms: “Antineoplastic Agents/pharmacology”, “Antineoplastic Agents/therapeutic use”, “Gossypium/chemistry: “Gossypol/analogues & derivatives”, “Gossypol/isolation & purification”, “Gossypol/pharmacology”, “Gossypol/therapeutic use”, “Neoplasms/drug therapy” and other keywords such as gossypol, cottonseeds, plant sources, anticancer properties, bioavailability of gossypol, studies in vitro and in vivo, antitumor action, and immunomodulatory effects have been used for the searching. The taxonomy of plants associated with gossypol was validated according to the World Flora Online [77] and chemical structures according to PubChem [51].

## Gossypol: general characterisation

### Natural sources of gossypol

Gossypol is a yellow crystalline pigment in the cotton plant seeds (*Gossypium sp*.) of the family Malvaceae [50]. The genus *Gossypium* consists of about 50 species, and the most cultivated species are *Gossypium hirsutum* and *Gossypium barbadense*. Gossypol is present mainly in the seeds but can also be found in the plant's roots, stems, and leaves. Gossypol is present in free form, and its primary function role is to protect the plant from pests and diseases. It bears noting that genetically modified cotton plants have a lower content of gossypol [62]. Gossypol is a polyphenol and a secondary metabolite detected in cotton plants belonging to the genus *Gossypium* (family *Malvaceae*). Its role in plants is crucial for development and self-protection [88]. Gossypol can be isolated from cottonseeds and by-products of the processing of cottonseed soap stock and cottonseed oil). It is yellow and is considered a hydrophobic substance with a limited water solubility [28]. Before gossypol was tested as an anticancer agent, it was studied in China as a male oral contraceptive. However, research was discontinued due to its side effects (such as hypokalaemia) and toxicity. In the late 1990s, the World Health Organisation (WHO) recommended that further research on gossypol as a male contraceptive drug should be abandoned [72]. Animal studies in rats and monkeys confirmed that both enantiomer forms are too toxic for male contraception [72]. The gossypol is mainly isolated from the seeds, but in some species, it can be found in other plant parts (roots, stem) [49]. Besides the species that belong to the genus *Gossypium*(*G. hirsutum, G. barbadense, G. arboreum, G. herbaceum, G. mustelinum*), gossypol can be isolated from the wood, leaves and flowers of *Thespesia populnea* [49]. Cotton plants were cultivated for thousands of years as textiles for clothing. The cotton fibres are almost pure cellulose [31]. Cotton plants are treated as annual cultivated plants because they form a small bush in the first year. After that, they can form large bushes or small trees. In this sense, *G. hirsutum*, also known as upland cotton or Mexican cotton, is the most planted cotton plant, and 90% of all cotton produced comes from this species. Another species is *G. herbaceum,* known as Levant cotton, the native species in Sub-Saharan Africa and Arabia. *G. barbadense* is a tropical, perennial cotton species that mainly grows as bushes or small trees with yellow flowers. Approximately 5% of the world's production is accounted for this species. Moreover, *G. arboreum* is known as tree cotton and is native to India, Bangladesh and Pakistan [47]. *Thespesia populnea* also belongs to the family *Malvaceae.* It is native to the tropical coast and is also known as the Portia tree, Pacific rosewood tree, Indian tulip tree or milo. In traditional medicine, different parts of this plant are used as antibacterial, anti-inflammatory, antinociceptive and hepatoprotective agents and for skin problems [58]. Plants are considered therapeutics due to the biosynthesis of secondary metabolites to which different pharmacological activities could be attributed. In this context, plant tissue cultures produce large amounts of secondary metabolites more efficiently than conventional methods [8, 59]. Analysing the expression of critical genes in the biosynthetic pathway of gossypol, Zhao et al. [88] have shown that the root system in vitro cultures of glanded and glandless plants is the most important system for gossypol production compared to other organs [87, 88]. Further studies confirmed the importance of hairy root cultures for gossypol production, as they can be used as bioreactors for increasing gossypol production. In this line, large amounts of gossypol could be produced upon infecting hairy root cultures of *G. hirsutum* with *Rhizobium rhizogenes* [66] or by *Agrobacterium rhizogenes*.w [70]. In vitro cultures of cottonseed embryos were used to develop plants with lower gossypol content in the seeds, which may benefit cotton breeders. These plants had increased gossypol levels in other organs to prevent pest occurrence [71].

### Traditional uses

In 1957, Chinese scientists reported collective infertility in Wang Village in China from the 1930s to 1940s [35]. This was due to replacing soybean oil with crude cottonseed oil for culinary purposes. Liu and colleagues [35] found that a biologically active substance in cottonseed oil, the polyphenol gossypol, caused male infertility. Antifertility effects have been demonstrated in mammals and birds *through *in vitro studies. In vivo experiments on animals and in vitro on testicular tissues have shown that gossypol reduces the production of testosterone [13, 20]. There is also evidence that this polyphenol can treat gynaecological diseases and disorders such as menorrhagia, endometriosis, and uterine fibroids. In addition, numerous primary and clinical studies have shown that gossypol has anti-tumour, antioxidant and immunomodulatory effects [28].

### Chemical and physical characteristics

**Fig. 1:**
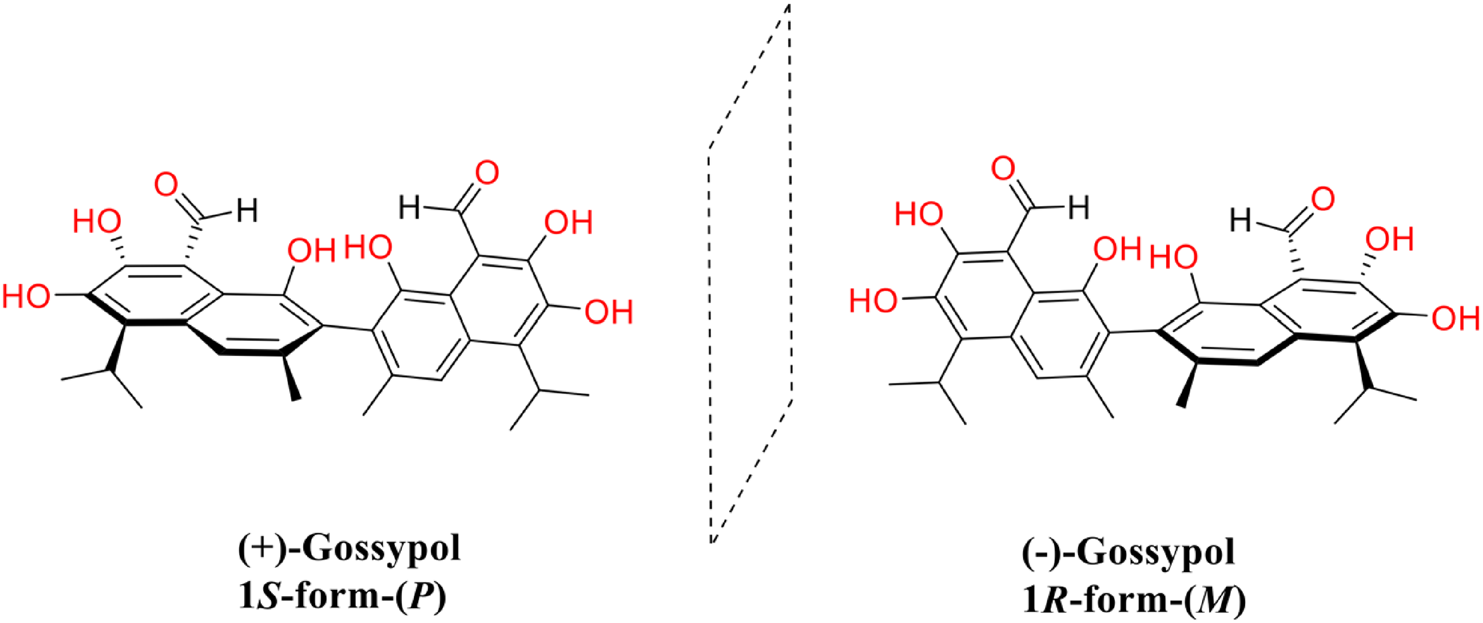
Structure of gossypol enantiomers: ( +)-gossypol and (-)-gossypol

**Fig. 2:**
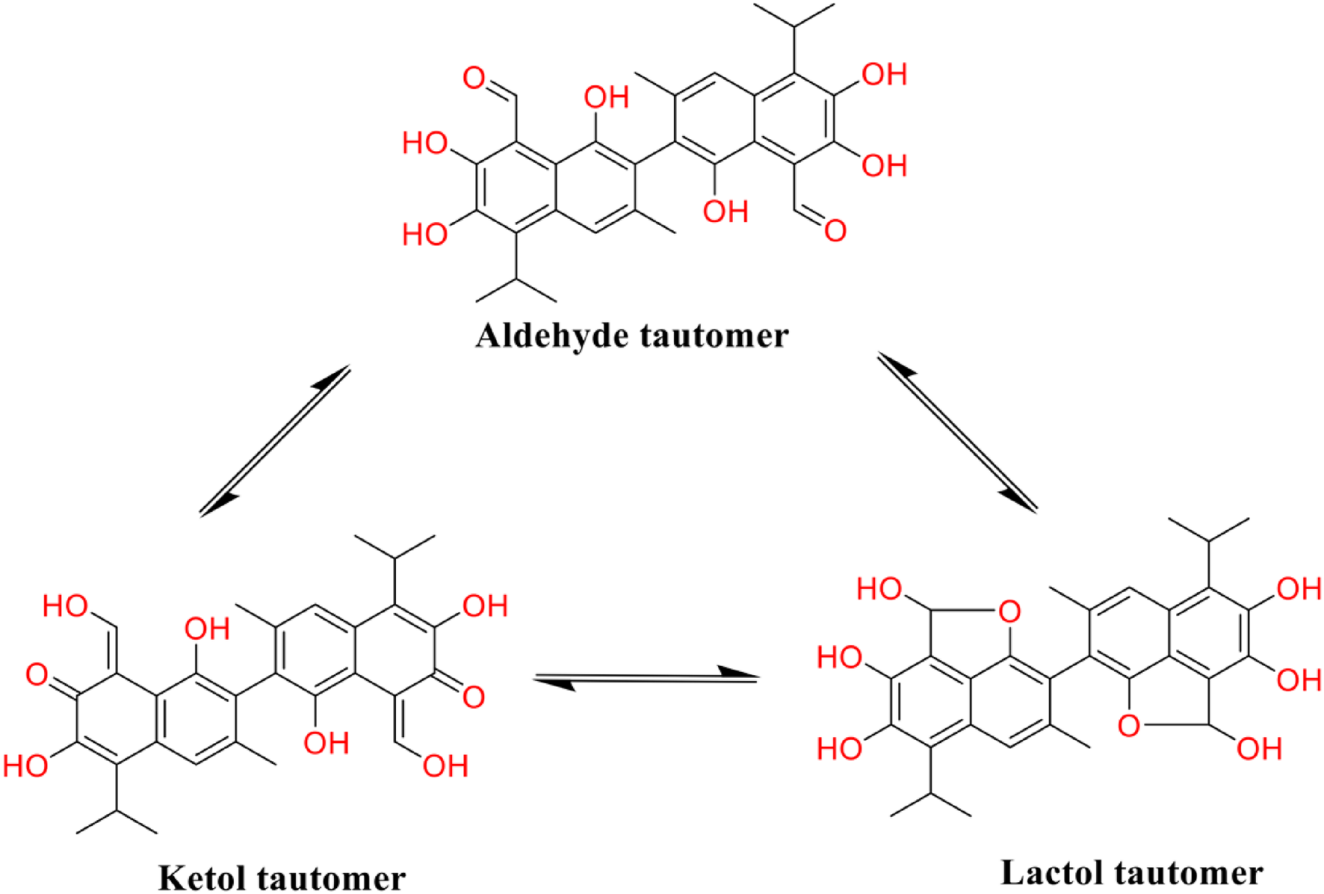
Chemical structure of tautomers of gossypol [38]

The molecular formula of gossypol is C_30_H_30_O_8,_ and its IUPAC name is 7-(8-formyl-1,6,7-trihydroxy-3-methyl-5-propan-2-ylnaphthalen-2-yl)-2,3,8-trihydroxy-6-methyl-4-propan-2-ylnaphthalene-1-carbaldehyde (PubChem database). Gossypol exhibits atropisomerism and exists as two enantiomers viz*.,* 1R form levo-gossypol ( −) and 1S form dextro-gossypol ( +) enantiomers [38] (Fig. 1). The bioactivity of ( −)-gossypol, also known as AT-101, is more potent than ( +)-gossypol and has been used in further research [85]. Different species of the genus *Gossypium* contain various dominant gossypol enantiomers in their plant organs [38]. There are three different tautomers of gossypol: aldehyde, ketone and lactol [38] (Fig. 2).

Naturally isolated gossypol consists of ( +)-enantiomer and (-)-enantiomer, but the gossypol (-)-enantiomer form, also known as AT-101, is recognised as more biologically active form and has been used in research [85]. Gossypol mainly occurs as gossypol, the two derivatives of acetic acid and formic acid. It bears noting that they possessed similar biological activities in basic and clinical studies [52].

### Semi-synthetic derivatives of gossypol

**Fig. 3:**
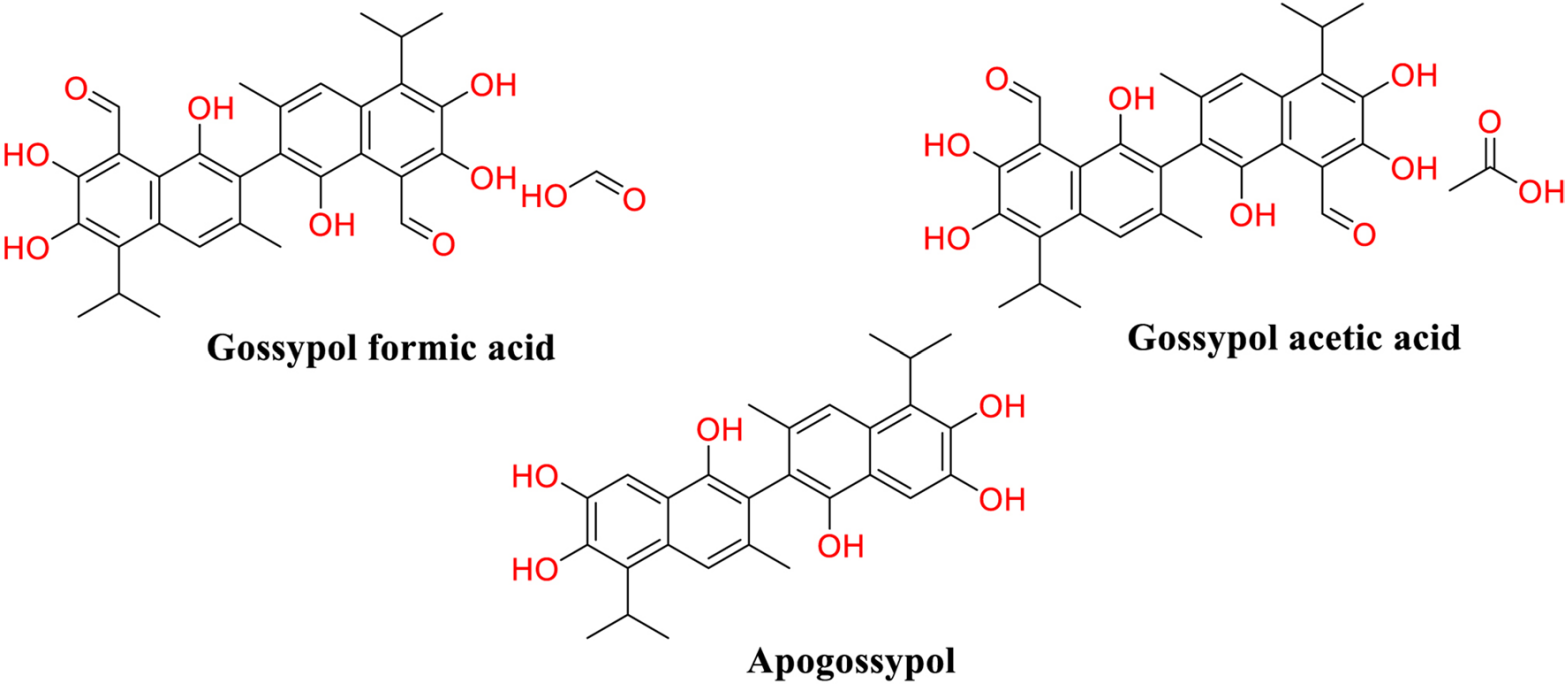
Structures of some gossypol derivatives

The landscape of gossypol derivatives has expanded significantly, with over 350 substances catalogued in the PubChem database, underscoring a broad interest in their diverse biological activities [51]. The gossypol derivatives usually include esters such as acetate, formate, metal complexes, and Schiff base derivatives [38] (Fig. 3). Beyond their initial recognition for antifertility effects, these derivatives have been discovered to possess other significant properties, including antiviral, immunomodulatory, and particularly, antitumor activities.

Among these derivatives, apogossypol, one of the earliest modifications of gossypol developed, has been reevaluated for its anticancer potential [14]. While it shares a similar pharmacokinetic profile with gossypol, apogossypol’s lower toxicity presents a more favorable therapeutic index [48]. Its mechanism, involving the cleavage of aldehyde groups from gossypol, may contribute to a differential interaction with the Bcl-2 family of proteins, pivotal regulators of apoptosis often dysregulated in cancer cells. Recent advancements in drug design have leveraged molecular cross-linking strategies to enhance the therapeutic efficacy of gossypol [65, 81]. For instance, the conjugation of gossypol with carboxymethyl cellulose, forming the drug Kagocel, illustrates a successful application of this approach. While Kagocel's antiviral efficacy is well-documented [2, 60], the concept of polymeric carrier linkage opens up new possibilities for cancer therapy. Another notable derivative is (S)-(-)-gossypol acetic acid, which has been explored for its dual mechanism of action: it not only induces apoptosis but also inhibits the proliferation of cancer cells by targeting kinases involved in cell cycle regulation; clinical trials have suggested that this compound may be effective against hormone-refractory prostate cancer, with manageable side effects [4]. R-(-)-gossypol, a natural enantiomer of gossypol, has been investigated for its pro-apoptotic activity, specifically its potential to target multiple myeloma [55]. The derivative shows promise in overcoming resistance to conventional therapies by simultaneously inducing apoptosis and inhibiting angiogenesis, a critical process for tumor growth and metastasis.

## Bioavailability of gossypol

Bioavailability refers to the extent and speed with which the active ingredient (drug or metabolite) enters systemic circulation. It also measures how much of a substance enters the bloodstream and reaches the target area [76]. The use of gossypol has some adverse effects, such as haemolytic anaemia diarrhoea, which can be avoided by using low drug dosage [19]. To determine if those side effects can be avoided with a low dose of gossypol, Gu et al. used three groups of male volunteers with different daily doses of the administered drug. They showed that a daily dose of 10 or 12.5 mg or 35 or a weekly dose of 43.75 mg of gossypol maintained contraceptive activity without side effects. Over the years of research, different approaches have been used to enhance the bioavailability and avoid the side effects of gossypol. Cho et al*.* [10] tried to use polymers with a drug combination including gossypol for the treatment of ovarian cancer, and the result showed reduced toxicity, but the drug encapsulation efficiency was low. Micelles poly(ethylene glycol)-block-poly(ε-caprolactone) (PEG-b-PCL) loaded with paclitaxel, cyclopamine, and gossypol were incorporated in vitro and in vivo models of human ovarian cancer and exhibited tumour growth inhibition and represent a method for the future treatment of ovarian cancer. Moreover, recent research focused on developing gastric floating sustained-release tablets of gossypol, thus enhancing its bioavailability and allowing controlled release with a reduction in hypokalaemia compared to gossypol powders [37]. To improve the water solubility and bioavailability of gossypol, Wang et al. [76] used gossypol-loaded pluronic F127 nanoparticles (GLPFNs), which increased bioavailability several times and exhibited higher organ uptake of the drug compared to using gossypol alone. About gossypol derivatives, it bears noting that apogossypol has a slower clearance rate than gossypol [49] with similar in vitro stability, while apogossypol hexaacetate has no oral bioavailability [26].

## Molecular mechanism of antitumor action of gossypol

Gossypol exerts its anticancer effects through a complex interplay of molecular mechanisms, leading to distinct biological consequences like apoptosis, autophagy, inhibition of tumor cell viability, angiogenesis, and immunomodulation; these mechanisms are intricately linked and often result in overlapping effects, contributing to the compound's overall antitumor activity.

### Apoptosis induction

#### Inhibition of anti-apoptotic proteins

The main mechanism of gossypol-anticancer activity is inducing apoptosis through suppressing anti-apoptotic proteins of the Bcl-2 family. This effect results from the inhibitory activity of AT-101, which acts as a mimetic agent to Bcl-2 Homology Domain 3 (BH3), downregulating Bcl-2-related proteins in human cancer cells.

#### Activation of apoptotic pathways

Also, it has been shown that gossypol may induce apoptosis via caspase-dependent and independent pathways. The caspase-dependent anti-tumour effect of gossypol is led by activation of caspase-3 and caspase-9. Apoptosis induced by independent pathways is made by alternations on the mitochondrial outer membrane permeabilisation [85].

#### Oxidative stress and mitochondrial dysfunction

**Fig. 4:**
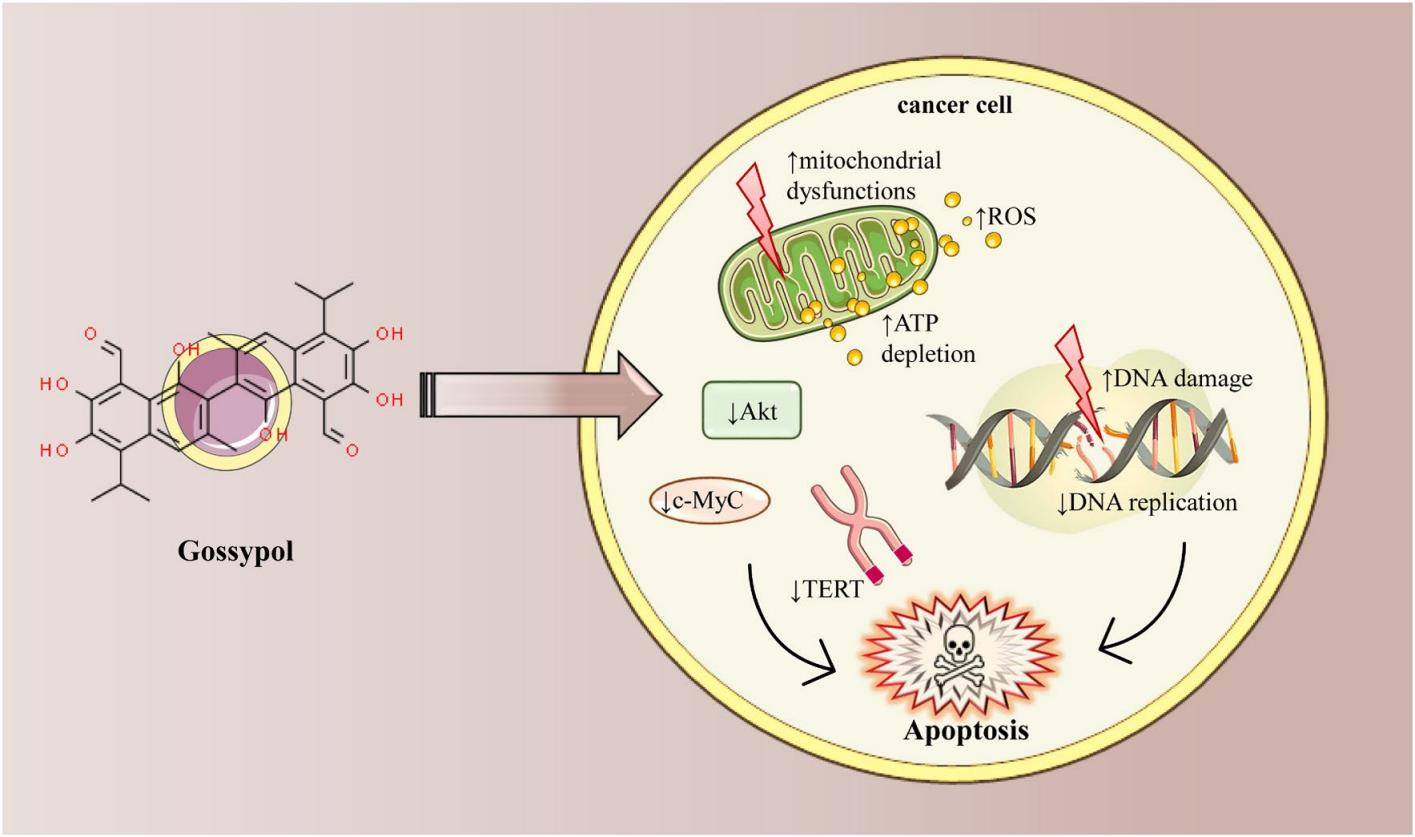
Apoptosis induction in cancer cells by gossypol. Gossypol interferes with cellular function by causing mitochondrial dysfunction, which leads to an increase in reactive oxygen species (ROS). This accumulation of ROS results in oxidative stress that damages cellular components, including DNA. Concurrently, gossypol's interaction with mitochondria leads to ATP depletion, crippling the cell’s energy supply and further exacerbating cellular stress. The compound also hinders key survival signals by downregulating Akt, a protein essential for cell survival, and c-Myc, a transcription factor that supports cell growth and proliferation. Additionally, gossypol inhibits the activity of telomerase reverse transcriptase (TERT), an enzyme vital for maintaining telomere length and thereby cell longevity. Together, these actions culminate in the activation of the cell’s apoptotic pathways, leading to programmed cell death. ↑ increase, ↓ decrease, telomerase reverse transcriptase (*TERT*), Adenosine triphosphate (*ATP*), Cellular myelocytomatosis oncogene (*c-MyC*), serine/threonine protein kinase (*Akt*)

Gossypol has been shown to induce also cell apoptosis through oxidative stress (Fig. 4). Gossypol treatment has been demonstrated to induce the production of reactive oxygen species (ROS) in tumour cells [80]. Elevated levels of ROS can trigger oxidative stress, DNA damage, and the activation of apoptotic pathways. In the case of multiple myeloma cells, treatment with 80 μmol/L gossypol resulted in a significant increase in cellular ROS levels, leading to ATP depletion, which induces mitochondrial dysfunction. The impaired function of mitochondria further contributes to the activation of apoptosis [80] (Fig. 4).

#### Epigenetic modulation and DNA damage

Recent studies indicated that gossypol may have epigenetic effects on human cancer cells. DNA damage can trigger apoptosis as a protective mechanism to eliminate cells with excessive genetic alterations [25]. Gossypol targets and damages nuclear DNA by upregulating DNA replication and mismatch proteins, among other effects [57]. Gossypol has been shown to block DNA synthesis in HeLa cells by inhibiting key nuclear enzymes, specifically polymerase alpha and polymerase beta. By inhibiting these enzymes, gossypol interferes with DNA replication, impairing DNA synthesis and potentially causing DNA damage [57].

#### Telomerase activity modulation

Gossypol has been found to modulate telomerase activity in leukaemia cells. Telomerase is an enzyme that plays a role in maintaining the length of telomeres, which are protective caps at the ends of chromosomes. Dysregulation of telomerase activity is commonly observed in cancer cells. Gossypol can modulate telomerase activity through both transcriptional downregulation and post-translational modification of telomerase reverse transcriptase (TERT). The transcriptional downregulation of TERT involves the inactivation of c-Myc, a transcription factor that regulates TERT expression. Additionally, gossypol can inhibit Akt, a signalling pathway involved in cell survival, leading to post-translational modification and inactivation of TERT. These effects on telomerase activity can ultimately result in the apoptosis of leukaemia cells [46].

#### Induction of autophagy as a complementary process of apoptosis

**Fig. 5:**
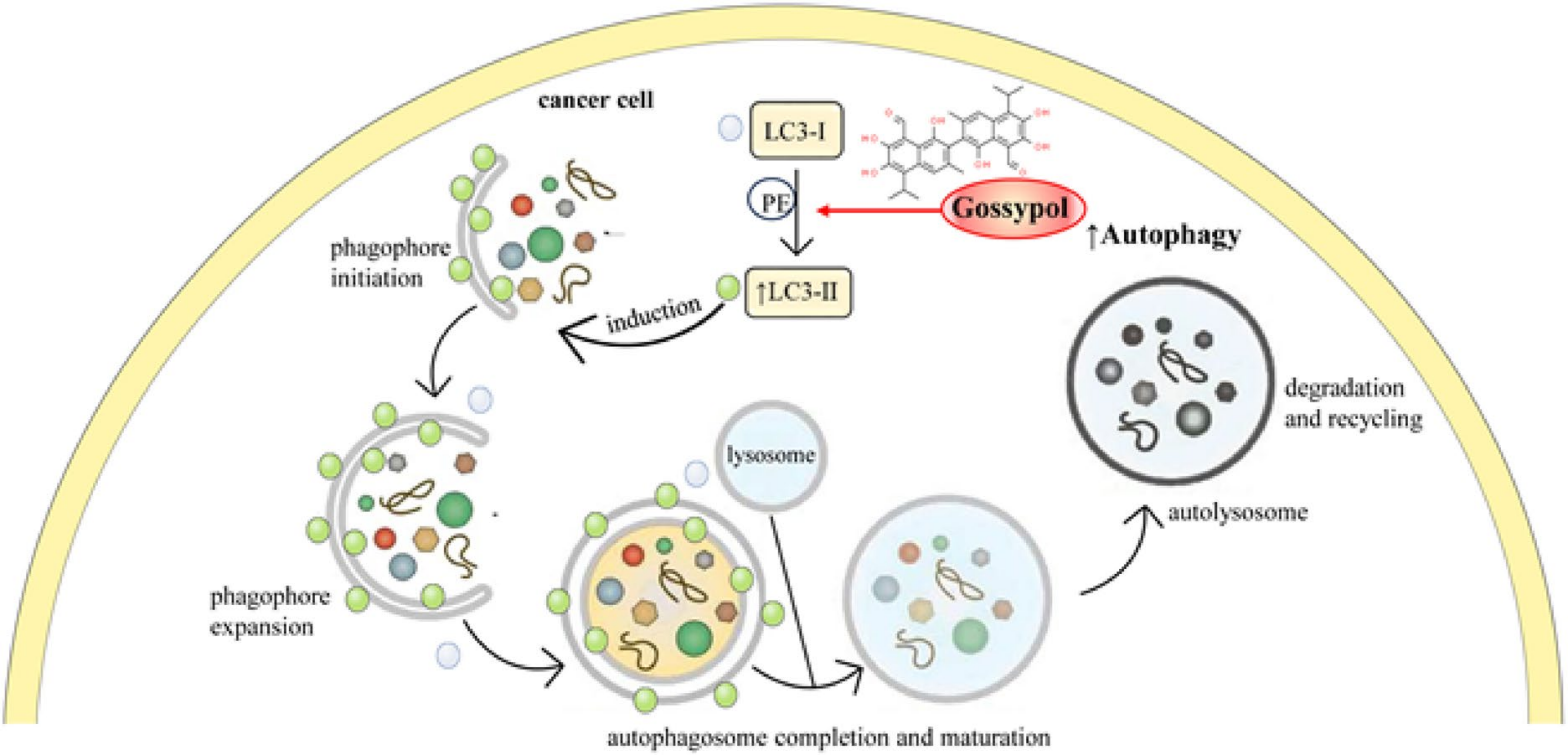
Autophagy induced by gossypol in cancer cells. Gossypol stimulates the conversion of LC3-I to its lipidated form LC3-II, which is a key step in autophagy initiation. LC3-II is associated with the autophagosome membrane. The process begins with the initiation of a phagophore, which expands to engulf cellular components targeted for degradation. The maturation of the phagophore leads to the formation of an autophagosome, which then fuses with a lysosome to form an autolysosome. Within the autolysosome, the encapsulated materials are degraded and recycled, providing the cell with a mechanism to remove damaged organelles and proteins. The action of gossypol in promoting this pathway suggests a potential therapeutic mechanism by which cancer cell survival is reduced through the enhanced turnover of cellular components. *LC3-I* Microtubule-associated proteins 1A/1B light chain 3B, form I, *LC3-II* Microtubule-associated proteins 1A/1B light chain 3B, form II, *PE* Phosphatidylethanolamine

Gossypol has been shown to induce autophagy, a cellular process involved in the degradation and recycling of cellular components (Fig. 5). Treatment of colorectal cancer cells with gossypol-induced autophagy and apoptosis [39]. The molecular mechanism is different for both, but eventually, their action is to remove unnecessary cells [40]. During autophagy, one of the well-known enzymes, LC3, is transformed from LC3-I to LC3-II. The treatment with gossypol in colorectal cancer cells increased the LC3-II/LC3-I ratio and induced autophagy [39]. On the other hand, in the same cell type, exposure to 20 and 40 µM gossypol significantly decreased Bcl-2 expression. Consequently, it led to increased expression of Bax, hence the release of Cyt-c and activation of caspase 3, which is the final step of apoptosis [39].

Figure 5 illustrates the role of gossypol in inducing autophagy within cancer cells, highlighting the conversion of LC3-I to the autophagosome-associated LC3-II through lipidation, and the subsequent steps leading to degradation and recycling of cellular components.

### Inhibition of tumor cell viability and signaling pathway modulation

**Fig. 6:**
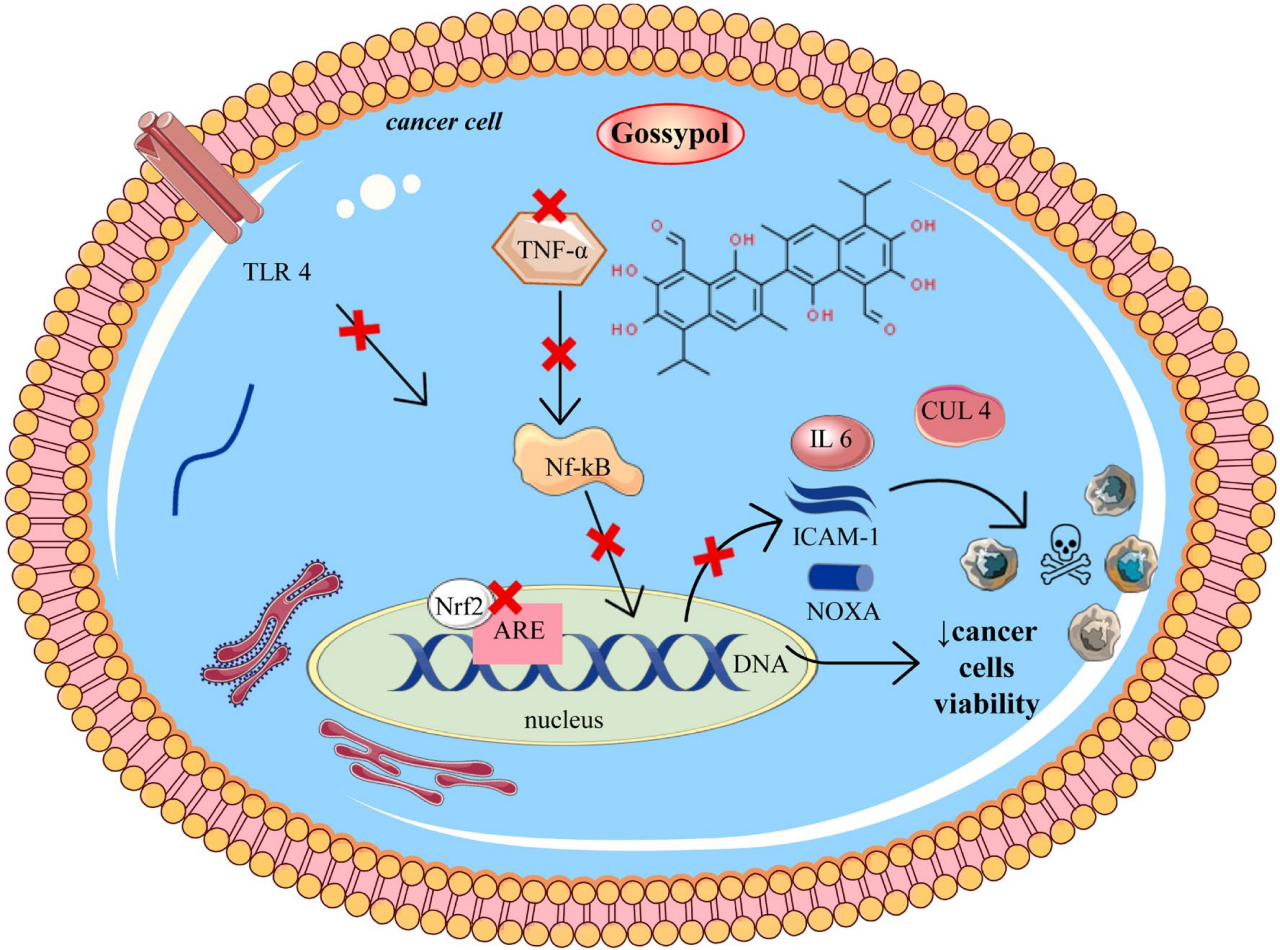
Illustrative diagram related to mechanisms of gossypol-induced decrease in cancer cell viability. The figure illustrates the multifaceted mechanisms by which gossypol reduces the viability of cancer cells. Gossypol inhibits TNF-α, which in turn prevents the activation of NF-kB, a transcription factor that regulates genes responsible for cell survival and proliferation. Additionally, gossypol disrupts the Nrf2/ARE pathway within the nucleus, leading to decreased DNA transcription of survival genes. It also inhibits the ICAM-1 pathway, contributing to reduced inflammation and interference with cancer cell adhesion. Gossypol's interaction with CUL4 appears to promote the degradation of survival proteins, further inducing cell death. Moreover, it suppresses NOXA, a pro-apoptotic protein, and induces the generation of ROS, leading to oxidative stress and damage. Collectively, these actions lead to a decrease in cancer cell viability. *ARE* Antioxidant Response Element, *CUL4* Cullin 4, ICAM-1 Intercellular Adhesion Molecule 1, IL-6 Interleukin 6, *NF-kB* Nuclear Factor kappa-light-chain-enhancer of activated B cells, *NOXA* Phorbol-12-myristate-13-acetate-induced protein 1, *Nrf2* Nuclear Factor Erythroid 2–Related Factor 2, *ROS* Reactive Oxygen Species, *TLR4* Toll-Like Receptor 4, *TNF-α* Tumor Necrosis Factor alpha. Symbols: ↓decrease, **X** inhibition

Recently, it has been shown that gossypol may act as an inhibitor of the Nrf2/ARE (nuclear factor erythroid 2–related factor 2/antioxidant-responsive element) signalling pathway in cancer cell lines. Nrf2 is a stress-activated transcription factor that binds to the promoter region of the ARE. This signalling pathway is recognised as a potential target for cancer chemotherapy. However, the over-activation of Nrf2 in cancer cells is also responsible for the chemotherapy resistance [29] in a study by Tang et al*.* [63], gossypol reduced Nrf2 protein stability, leading to the inhibition of the Nrf2/ARE pathway, resulting in a significant decrease of cell viability in human cancer cells and stimulation of cytotoxicity in chemo-resistant cancer cell lines. In cancer cells, tumour necrosis factor-alpha (TNF-α) can stimulate the expression of intercellular adhesion molecule-1 (ICAM-1) through the activation of nuclear factor-kappa B (NF-κB). ICAM-1 is involved in cell adhesion processes and plays a role in inflammation [53]. Treating breast cancer cells with gossypol has been shown to block the binding of NF-κB to the promoter regions of ICAM-1, suppressing TNF-α-induced ICAM-1 expression. This indicates that gossypol inhibits the NF-κB signalling pathway, which prevents the stimulation of ICAM-1 by TNF-α [45]. Gossypol has demonstrated similar inhibition of cancer cells viability, apoptosis and inflammatory activity in chondrocytes. It down-regulates the expression of CX43, nuclear NF-κB, TNF-α, toll-like receptor 4 (TLR4), and interleukin-6 (IL-6) in these cells, indicating its potential anti-inflammatory and anti-apoptotic effects [33]. In several human cancer cell lines, gossypol has been found to block the neddylation of cullin enzymes (CUL5 and CUL1) by directly binding to the SAG-CUL5 or RBX1-CUL1 complex. This leads to the accumulation of both the pro-apoptotic protein NOXA and the anti-apoptotic protein MCL1, suggesting a complex modulation of apoptosis by gossypol [82]. Another study in colon cancer cell lines DLD-1 and COLO 205 indicated that gossypol significantly reduced the invasion, migration, and adhesion of these cancer cells by suppressing the FAK pathway and ETM [24]. Figure 6 summarizes the illustrative scheme related to the mechanisms of gossypol-induced decrease in cancer cell viability .

### Inhibition of angiogenesis in tumor cells

**Fig. 7:**
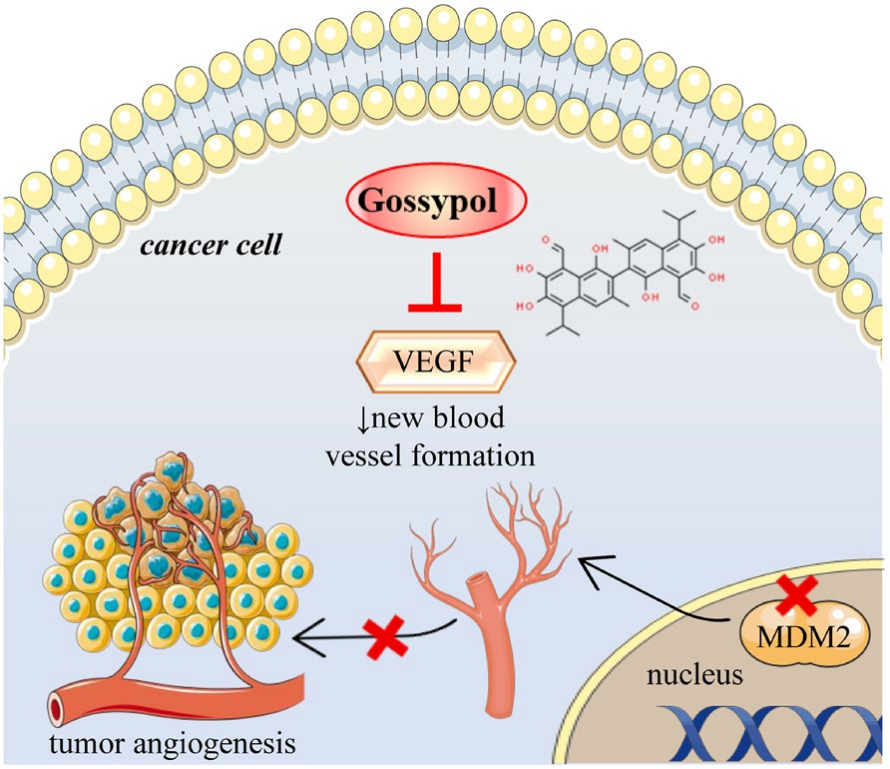
Inhibition of angiogenesis in tumor cells by gossypol.  It exerts this  therapeutic effect by downregulating the expression of vascular endothelial growth factor (VEGF), a critical protein that stimulates the formation of new blood vessels (angiogenesis) within tumor tissues. The suppression of VEGF leads to a decrease in new blood vessel formation, effectively starving the tumor of the necessary nutrients and oxygen needed for growth. Additionally, gossypol interferes with the MDM2 protein within the nucleus. MDM2 is known to negatively regulate the tumor suppressor p53, and by inhibiting MDM2, gossypol may contribute to the reactivation of p53's tumor-suppressive functions. Through these mechanisms, gossypol effectively inhibits tumor angiogenesis, contributing to its anticancer effects. *VEGF* Vascular Endothelial Growth Factor, *MDM2* Mouse Double Minute 2 Homolog

In a recent study, after the treatment with gossypol, the binding between the MDM2 protein and VEGF mRNA was disrupted in breast cancer cells [78]. As a result, the expression of MDM2 and VEGF proteins is significantly decreased. MDM2 is an oncoprotein that plays a role in inhibiting the tumour suppressor function of p53. At the same time, VEGF is involved in promoting angiogenesis, forming new blood vessels to supply nutrients to tumours [78]. The disruption of the MDM2-VEGF mRNA binding by gossypol leads to a decrease in the translation of VEGF, which subsequently affects MDM2 protein levels. This decrease in MDM2 protein can have dual effects [78]. First, it can promote cancer cells death, as MDM2 inhibits the tumour suppressor function of p53, and a reduction in MDM2 levels may allow for p53-mediated apoptosis to occur. Second, the decrease in VEGF translation leads to anti-angiogenic effects, as VEGF is a critical factor in promoting the formation of new blood vessels. The disruption of MDM2-VEGF mRNA binding by gossypol results in decreased MDM2 and VEGF protein expression, which can contribute to both cancer cells death and anti-angiogenesis in breast cancer [78].Fig. 7 illustrates the anti-angiogenic mechanism of gossypol in cancer cells (Fig. 7).

### Immunomodulatory effect

Treatment with gossypol has been found to increase the expression of HLA-I/II molecules [80]. HLA molecules, also known as human leukocyte antigens, play a crucial role in the immune system by presenting antigens to immune cells and activating immune responses. The upregulation of HLA-I/II molecules after gossypol treatment suggests a modulation of the cellular immune system [80]. Increased expression of these molecules can enhance antigen presentation and recognition by immune cells, such as T cells and natural killer (NK) cells. This modulation of the immune system may have several implications for anti-cancer activity. Firstly, the increased expression of HLA-I/II molecules can enhance the recognition of tumour cells by cytotoxic T lymphocytes (CTLs) and NK cells [56]. This, in turn, can lead to enhanced immune surveillance and the elimination of cancerous cells. Secondly, the upregulation of HLA-I/II molecules can also facilitate the presentation of tumour-specific antigens to immune cells, activating specific immune responses against cancer cells [56]. This immune modulation may contribute to its anti-cancer activity by enhancing immune recognition and response against tumour cells [80]. These findings highlight the mechanisms of action of gossypol in cancer cells, including the modulation of signalling pathways involved in cell adhesion, inflammation, and cancer cells death. The ability of gossypol to interfere with these pathways makes it a promising candidate for further exploration in the development of anticancer therapies.

Table 1 presents a detailed overview of Gossypol's antitumor mechanisms and its impacts on cancer cells, delineating the key mechanistic actions and associated cellular responses.

**Table 1:** Overview of gossypol’s antitumor mechanisms

Anticancer effect	Mechanistic actions	Impact on cancer cells	References
Apoptosis induction	Inhibits anti-apoptotic proteins (Bcl-2 family); ↑caspase-dependent/independent pathways	Induces apoptosis in various cancer cell types	[85]
Oxidative stress and mitochondrial dysfunction	↑ ROS, leading to DNA damage and mitochondrial dysfunction	Leads to cell apoptosis through oxidative stress	[80]
Epigenetic modulation and DNA damage	↑ DNA replication/mismatch proteins; ↓ polymerases α/β	Triggers apoptosis due to DNA damage	[25, 57]
Telomerase activity modulation	Modulates telomerase activity; Affects TERT through transcriptional downregulation/post-translational modification	Results in apoptosis of leukemia cells	[46]
Induction of autophagy	Transforms LC3-I to LC3-II; ↓ Bcl-2 and ↑ Bax expression	Induces autophagy, leading to apoptosis	[39, 40]
Inhibition of tumor cell viability and signaling pathway modulation	Inhibits NRF2/ARE pathway; ↓ cell viability, stimulates cytotoxicity in resistant cancer cell lines	Reduces viability and resistance in cancer cells	[24, 53, 63, 82]
Inhibition of angiogenesis	Disrupts MDM2-VEGF mRNA binding; ↓ MDM2/VEGF protein expression	Prevents angiogenesis, affecting tumor growth	[78]
Immunomodulatory effect	↑ HLA-I/II molecule expression; Enhances antigen presentation and immune response	Enhances immune-mediated recognition and elimination of cancer cells	[56, 80]

*ARE* Antioxidant-Responsive Element, *Bax* BCL2 Associated X Protein, *Bcl-2* B-Cell Lymphoma 2, *HLA-I/II* Human Leukocyte Antigen Class I/II, *LC3* Microtubule-associated proteins 1A/1B light chain 3B, *MDM2* Mouse Double Minute 2 homolog, *NF-κB* Nuclear Factor kappa-light-chain-enhancer of activated B cells, *NRF2* Nuclear Factor Erythroid 2–Related Factor 2, *ROS* Reactive Oxygen Species, *TERT* Telomerase Reverse Transcriptase, *VEGF* Vascular Endothelial Growth Factor. Symbols: ↑: Indicates an increase or upregulation; ↓: Indicates a decrease or downregulation

## Pharmacological studies: underlying molecular mechanisms and targets in various types of cancers

Since 1984, the first in vitro study of gossypol that indicated its antitumour potential [69], numerous in vitro and in vivo studies of gossypol have demonstrated a wide range of anticancer activity and mechanisms of action.

### Head and neck carcinoma

A study done by Benvenuto et al*.* showed that racemic gossypol induced apoptosis and autophagy in head and neck carcinoma (HNC) cell lines [carcinoma of the tongue (CAL-27), pharynx (FaDu) or salivary gland (A253)] [1]. The effect of gossypol on cell proliferation was time- and dose-dependent and significantly decreased cell survival after 48 and 72 h with doses of 5–80 µM, while doses of 10–80 µM gained the same effect after 24 h. In the same study, an in vivo investigation was performed using BALB/c mice subcutaneously injected with SALTO cells (neu-overexpressing salivary gland cancer cells). After 3 weeks, the mice were treated with an intratumoural injection of gossypol weekly for 2 weeks and then received an oral dose of gossypol 3 times a week. Gossypol reduced tumour growth and prolonged median survival time.

### Breast cancer

Previous in vitro studies indicated that gossypol possesses anticancer activity on several cancer cell lines. Treatment of human breast cancer cells (MCF7) with different concentrations of gossypol (up to 100 µM) significantly decreased cell growth after 24 h [5]. The same treatment of pancreatic cancer cells (MIA PaCA-2) showed that cell viability was significantly reduced after 2 h of treatment [5]. In another study by Xiong et al*.* [78] on human breast cancer cells (MCF-7, MDA-MB-231, MDA-MB-468, ZR-75-1, and T47D), two molecules were investigated as targets for the anti-tumour effects of gossypol viz*.*, MDM2 (Mouse Double Minute 2) which is an RNA-binging protein and its target vascular endothelial growth factor (VEGF) mRNA. Both molecules (MDM2 and VEGF), responsible for tumour progression, were inhibited by gossypol in a time- and dose-dependent manner (up to 24 h and up to 10 µM). In addition, gossypol promoted apoptosis in all breast cancer cell lines. In the same research, an in vivo study was done on mice using xenograft models (MCF-7 and MDA-MB-468) treated intraperitoneally, where the dose was 10 mg/kg/day of gossypol for 4 weeks. In both types of xenograft models, inhibition of tumour growth by gossypol was observed [78]. In a study with triple-negative breast cancer cell lines (MDA-MB-231 and MDA-MB-468), gossypol decreased cancer cell viability by suppressing the expression of the critical chemokines, CCL2 in MDA-MB-231 cells and IL-8 in MDA-MB-468 [42].

### Lung cancer

The in vitro study on non-small cell lung cancer NSCLC cell lines (H1975) confirmed that gossypol (up to 20 µM for 24 h) inhibits cell proliferation and cell migration and induces caspase-dependent cell apoptosis [74] in these cancer cells. Also, it was reported in the same study that gossypol achieves this anti-tumour effect by targeting EGFR^L858R/T790M^. Another survey of NSCLC cell lines indicated that gossypol treatment may overcome the EGFR tyrosine kinase inhibitors (EGFR-TKIs) resistance in these cells by targeting EGFR^L858R/T790M^ and YAP/TAZ (part of Hippo signalling pathway) [79].

The newly reported mechanism of gossypol's anti-tumour effect is the inhibition of cullin neddylation. In vitro study on human lung cancer cell lines (H1299 and H358) confirmed this finding in a dose-dependent manner [82].

### Digestive cancers

#### Esophageal cancer

In a study by Song and colleagues [61], AT-101 were analysed in vitro, in vivo*,* and in a pilot clinical trial targeting cancer stem cells and patients with gastroesophageal carcinoma. In vitro studies used gastroesophageal cancer cell lines, including several esophageal cells (EC) and gastric cell (GC) lines. AT101 showed cell growth inhibition in a dose-dependent manner (up to 10 µM, treatment 3 and 6 days) in all four GC (AGS, KATO III, SNU1, GT-5) cell lines. In an analysis of the anti-tumour mechanism, AT-101 showed a strong inhibitory effect on YAP1 in the Hippo signalling pathway and SOX9 (SRY-Box Transcription Factor 9). YAP1 and its target SOX9 were up-regulated significantly in both OC and GC compared to normal tissues.

The effect of AT-101 was also investigated in combination with Docetaxel, the standard treatment for gastroesophageal cancers. The combination treatment of AT-101 and Docetaxel showed a synergistic effect in inducing apoptosis by down-regulating YAP1, SOX9 and β-catenin and BCL-2/MCL-1 in GC cells. In contrast, the main mechanism in EC cells, which normally have low expression of BCL-2, depended more on the inhibition of YAP1/SOX9.

In vivo studies with nude mice bearing esophageal squamous cell carcinoma JHESO cell xenografts showed reduced tumour volume and weight after treatment with AT-101. The female null mice were injected subcutaneously with JHESO cells. When the tumour volume reached 50 mm^3^, the mice were divided into four groups: control, AT-101-only (7.5 mg/kg daily, p.o., 5 days/week), Docetaxel-only (1 mg/kg, i.p., weekly injection), and combination Docetaxel (1 mg/kg, i.p., weekly injection) plus AT-101 (7.5 mg/kg daily, p.o., 5 days/week). Three weeks later, all mice were sacrificed, and the most significant reduction in tumour volume and growth was observed in the group treated with the combination of Docetaxel and AT-101. A substantial decrease in YAP1/SOX9 levels was detected in the same group, confirming the synergistic effect previously observed in vitro by the same mechanism. However, the best result was gained when a single irradiation of 10 Gy for 90 s was administered during the second week of the 3 week treatment with the combination of Docetaxel and AT-101.

#### Pancreatic cancer

A recent study using pancreatic cancer cell lines (BxPC-3 and MIA PaCa-2) confirmed the antitumour effect of gossypol by triggering the endoplasmic reticulum stress-related PERK-CHOP signalling pathway [32]. Gossypol induced mitochondrial apoptosis by increasing caspase-3 levels.

#### Hepatic camcer

In research done by Mayer et al*.* [41] on human-derived hepatoma (HepG2) and colon carcinoma (HCT-116) cell lines, a short treatment (6 h) with gossypol stimulated hyperacetylation of histone protein H3 and/or tubulin. Prolonged incubation with gossypol (up to 96 h) with different concentrations (5–50 µM) significantly reduced cell viability and proliferation of hepatoma (HepG2, Hep3B) and colon carcinoma (HCT-116, HT-29) cells in a time- and concentration-dependent manner due to caspase 3/7 activity. However, in the same research, it was showed that gossypol has potentially toxic effects on non-malignant cell lines at concentrations higher than 5 µM, as well as embryotoxic effects at concentrations higher than 2.5 µM [41].

#### Colon cancer

In another study with human colon cancer cells (COLO 225), gossypol significantly reduced cell viability in a time and dose-dependent manner [6]. Moreover, the effect of gossypol treatment on the mRNA level of 55 genes was analysed. It was shown that the expression of most genes was suppressed by high levels of gossypol (up to 100 µM) [5].

### Cervical cancer

Treatment of human cervical cancer cells (HeLa and SiHa cell lines) with gossypol (up to 10 µM for 48 h) showed a strong inhibitory effect on migration and invasion by targeting the focal adhesion kinase (FAK) signalling pathway and reversing TGF-β1-induced epithelial-mesenchymal transition (ETM) [23]. In the same study as the in vivo model, 15 immunodeficient female BALB/c AnN nude mice were used. All mice were injected subcutaneously with SiHa cells and divided into one control and two treatment groups. In the treatment groups, the mice were fed 5 times per week with gossypol 10 mg/kg and 20 mg/kg via oral gavage. The subsequent intraperitoneal administration of D-luciferin was used for visualisation. Also, some studies analysed pulmonary metastasis in SCID (Severe Combined Immuno Deficiency) mice using the same protocol. The animal model study showed that gossypol (especially 20 mg/kg) significantly reduced tumour growth and average tumour size after 40 days [23].

### Prostate cancer

Another possible mechanism of gossypol’s anti-tumour effect suggests that it may be used to inhibit androgen formation in prostate cancer cells. Gossypol acetate significantly inhibits rat’s 5α-reductase 1 and 3α-hydroxysteroid dehydrogenase [7].

Table 2 summarizes the molecular mechanisms and anticancer effects of gossypol in various cancer models.

**Table 2:** Pharmacological studies of gossypol: molecular mechanisms and anticancer effects in various cancer models

Type of study	Cancer cell line/animal model	Tested concentrations/doses	Effects/ mechanisms	Ref.
In vitro In vivo	HNC cell lines (CAL-27, FaDu, A253); BALB/c mice with SALTO cells	5–80 µM (in vitro); intratumoral and oral (in vivo)	↑apoptosis autophagy induction	[1]
In vitro In vivo	Breast cancer cells (MCF7, MDA-MB-231, MDA-MB-468, ZR-75-1, T47D); MCF-7 and MDA-MB-468 xenografts in mice	100 µM (in vitro); 10 mg/kg/day (in vivo)	decreased cell growth; ↓MDM2, ↓VEGF apoptosis promotion	[78] [5]
In vitro	NSCLC cell lines (H1975)	20 µM	-inhibition of cell proliferation and migration; caspase-dependent apoptosis	[74]
		80 µM	overcoming EGFR-TKIs resistance; targeting EGFRL858R/T790M and YAP/TAZ	[79]
In vitro	Human lung cancer cell lines (H1299, H358)	0.1–50 µM	inhibition of cullin neddylation	[82]
In vitro, In vivo, Clinical study	Gastroesophageal cancer cell lines; nude mice with JHESO cell xenografts	10 µM (in vitro); 7.5 mg/kg (in vivo)	growth inhibition; downregulation of YAP1, SOX9; combination treatment synergy	[61]
In vitro	Pancreatic cancer cell lines (BxPC-3, MIA PaCa-2)	200 µM	mitochondrial apoptosis via PERK-CHOP signaling	[32]
In vitro	Hepatoma (HepG2, Hep3B) colon carcinoma (HCT-116, HT-29) cells	5–50 µM	reduced cell viability; ↑caspase 3/7 activity	[41]
In vitro	Human colon cancer cells (COLO 225)	100 µM	reduced cell viability; gene expression modulation	[6]
In vitro In vivo	Cervical cancer cells (HeLa, SiHa); BALB/c AnN nude mice with SiHa cells	10 µM (in vitro); 10–20 mg/kg (in vivo)	inhibition of migration and invasion; tumor growth reduction	[23]
In vitro	Prostate cancer cells	100 µM	inhibition of androgen formation; 5α-reductase and 3α-hydroxysteroid dehydrogenase inhibition	[7]

## Clinical studies

The gossypol in the form of gossypol acetate tablets is available on the drug market for tumour treatment in China [85]. However, in the rest of the world, especially in the USA, the anti-tumour activity of gossypol is currently under clinical trial investigation. Clinical trials that investigated AT-101 ((-)-gossypol) are available on the website https://clinicaltrials.gov (accessed in March 2023). Most clinical trials were completed, and most were performed in the USA. All currently available clinical trials are phase I/II, with only one trial phase III.

In this sense, Song et al. included 13 patients with gastroesophageal cancer in an open-label clinical trial phase I/II clinical pilot study [61]. They received AT-101 (p.o., 5 days/week) along with chemotherapy (Docetaxel, 20 mg/m^2^ as bolus once a week) and radiation (50.4 Gy in 28 fractions). The first seven patients received a dose of 10 mg/day of AT-101, and when dose-limiting toxicity was not observed, the following 6 patients received a dose of 20 mg/day. A total of 9 serious adverse events (SAE) were reported, none of which were not correlated to AT-101. Among the adverse events (AE), vomiting, anorexia and odynophagia were most commonly reported. The phase I clinical trial indicated that patient survival was longer than expected, but phase II was terminated early by the sponsor's decision.

In phase II clinical trial performed by the National Cancer Institute (NCI) in the USA from 2008 to 2012, the effect of gossypol was investigated in the treatment of patients with progressive or recurrent glioblastoma multiform (https://clinicaltrials.gov/ct2/show/NCT00540722). In total, 56 adult patients were enrolled in the open-label, single-arm trial. Patients received oral AT-101 once daily for 21 days, and treatment was repeated every 28 days without disease progression or unacceptable toxicity. Related to SAE, six cases were reported, per one case in the cardiac, gastrointestinal, nervous systems, metabolism disorders and two cases of fatigue. In 50% of patients, AE and fatigue were the most commonly reported AE (37.5%). However, the results of the study have yet to be available. In a randomised, double-blind, placebo-controlled multicentre phase III clinical trial performed between January 2014 and February 2017, gossypol acetate tablets (20 mg/tablet) produced by Xi’an Northern Pharmaceutical Co., Ltd were tested with a placebo in 102 patients with NSCLC [75]. The patients were divided into a control and an experimental group. In the experimental group (n = 50), patients received 75 mg/m^2^ Docetaxel and 75 mg/m^2^ Cisplatin on day 1 with 20 mg gossypol once daily for 14 days every 21 days. The control group (n = 52) received the same standard treatment with placebo tablets instead of gossypol acetate tablets. All patients received 5-hydroxytryptamine receptor antagonists and corticosteroids on chemotherapy's first and second days. Due to a lack of compliance, 40 patients were lost in the follow-up phase and excluded from the final analysis (19 in the experimental group and 21 in the placebo group). Overall, gossypol was well tolerated, without signs of toxicity, and no SAE was recorded related to drug treatment. No statistically significant difference was found between the experimental and placebo groups in progression-free survival (PFS) and overall survival (OS). However, owing to the small group size, median PFS (mPFS) and median OS (mOS) were calculated as the experimental group had better outcomes of increased mPFS 2.53 months longer) and mOS (increased by 3.67 months) than the placebo group.

## Other biomedical properties of gossypol

In the middle of the last century, an early study was conducted on the properties of gossypol to prevent the lipid peroxidation of carotene [22]. There are many studies about the antioxidant properties of gossypol. One of them showed inhibiting microsomal peroxidation in the liver of rats [30]. Another study also confirmed the antioxidant properties of gossypol in hepatic cells of male rats, in which the concentration of reduced glutathione was significantly decreased, indicating a lowering in the levels of peroxides and free radicals [15]. In this line, a study proved that gossypol is an effective inhibitor of oxidative stress-induced necrosis in retinal pigment epithelial cells where the antioxidant activity of gossypol was compared with the usual concentration of other commonly used antioxidants such as α-tocopherol and ascorbic acid. Gossypol proved a potent inhibitor of oxidative stress-induced death of retinal pigment epithelial cells and showed an optimal biological protective effect in a low concentration range [21]. Gossypol is an effective oxidative stress reliever and a powerful antioxidant, as it can prevent DNA damage caused by hydrogen peroxide by scavenging free radicals in a dose-dependent manner. Besides, it can reduce the ferric ions [73].

Gossypol has shown potent antiviral activity against HIV-1 in peripheral blood mononuclear cells by inhibiting reverse transcriptase [34]. In addition, the significant potential of gossypol in malaria therapy was confirmed after testing gossypol and 13 other associate derivatives on two strains of *Plasmodium falciparum* [54]*.* The other antiparasitic activity of gossypol was investigated against *Entamoeba histolytica* and *Trypanosoma cruzi,* where gossypol binds to essential ameobic proteins and thus inactivates the life cycle of this parasite [18]. In the other strain, *Trypanosoma cruzi*, the parasite was completely immobilised after the treatment with 100 mM gossypol by inhibition of α-hydroxy acid dehydrogenase and malate dehydrogenase [44].

Furthermore, the male contraceptive potential for gossypol is considered one of the best-known biological activities of gossypol. Different in vivo animal models have confirmed the contraceptive potential of gossypol where the antifertility effect of gossypol investigated in the male reproductive system of mice, rats, dogs, and monkeys showed that different products of gossypol metabolism deposited in the cauda epididymis and vas deferens causing inhibition of spermatogenesis in the testis and preventing sperm maturation in the epididymis [64]. In a study through oral administration of different oral doses of gossypol from 5 to 10 mg/kg/day for 12 weeks in male hamsters and rats with induced sterility. At the higher dose, gossypol in male rats significantly reduced the production of sperm [9]. Gossypol in a µM dose reversibly inhibits the following acrosomal enzymes: acrosin, azocoll proteinase, neuraminidase, hyaluronidase, and arylsulfatase [84] or decreases the levels of potassium ions (K^+^) in sperm membrane [68].

Moreover, the treatment of boars with gossypol inhibited glycolysis and the respiratory chain, leading to a decrease in oxidative phosphorylation and adenosine triphosphate (ATP) synthesis, causing a decrease in energy supply and inhibition of sperm motility [67]. Gossypol has proven to be a reliable contraceptive for men, as confirmed by research conducted in the 1970s by a team of researchers from the Institute of Pharmacology of the Chinese Academy of Medical Sciences on 10,000 male volunteers. A pill containing 20 mg/kg gossypol per day was found to have a contraceptive effect on men without affecting hormonal balance, weight, blood pressure, or the main biochemical parameters (1978, [27]. Those extensive and long-term studies of oral regimens of gossypol as a male contraceptive evaluated the potential side effects like hypokalaemia, an increase of alkaline phosphatase and follicle-stimulating hormone. All this prompted an additional study involving subjects from Latin America, Africa, and Asia to evaluate the efficacy and safety of gossypol. The lower dose of gossypol (10–15 mg/day for 12–16 weeks) was consistent with the contraceptive properties of this pili, without drug-attributable adverse events, with a reversible sperm count 52 weeks after stopping treatment [11]. Not surprisingly, the contraceptive efficacy of gossypol was reported to be over 99% in several studies [12, 36].

The behavioural study on animal models suggests that rats have an aversion to voluntary ethanol drinking because a metabolic reaction between gossypol and alcohol inhibits hepatic alcohol dehydrogenase. Therefore, it increases the development of condensation products between the biogenic precursor amine and the unreacted aldehyde intermediate(s) to form alkaloid-like compounds (Messiha, 1991).

## Toxicity, safety and side effects

Gossypol has toxic properties, and hence, it aids in the protection of cotton plants from several insects and/or pathogens. In this sense, animal feed cotton meals could have toxicity on the long-term feed. Besides, it can be a source of human toxicity directly or through the food chain [38]. Free gossypol may cause anorexia, respiratory distress, impaired weight gain, apathy, immunity impairment, cell and blood vessel damage, and heart failure and may lead to death. The main toxicity is male infertility, which could be irreversible and hypokalaemia [16]. Among the main side effects of gossypol noticed during clinical studies are haemolytic anaemia, diarrhoea, and other gastrointestinal-related symptoms [55]. In this sense, there are several methods for cotton meal physical detoxification, viz*.,* dry heating, immersion, puffing, and separation by centrifugation. Chemical detoxification includes extraction, oxidation by oxidising agents, and alkali immersion. Besides, microbial fermentation could reduce free gossypol toxicity [38]. Moreover, the noticed side effects of gossypol could be managed by decreasing the doses and treating symptoms whenever possible [55]. In this context, the derivatisation of gossypol can lead to better biological potentials alongside a lower toxicity [17, 86].

## Limitations and future perspectives

The journey of gossypol in medical applications, especially as an anticancer agent, is not without its hurdles. The narrow therapeutic range of gossypol, coupled with significant concerns such as the risk of irreversible sterility and severe side effects like hypokalemia, has restricted its widespread acceptance in clinical settings [3, 72, 83]. These challenges are further compounded by the cytotoxic nature of gossypol and its derivatives, attributed to the phenolic oxygen atoms. Thus, the need to balance efficacy with safety remains a critical area of ongoing research. Despite these limitations, gossypol continues to stand out as a potential natural anticancer agent. Years of research have elucidated its various mechanisms of action, and its efficacy has been demonstrated in numerous in vitro and in vivo studies. The addition of about 25 phase I and II clinical trials and its availability in the Chinese drug market further underscore its therapeutic potential. Addressing the toxicity concerns, there is a growing interest in developing new gossypol derivatives with minimal side effects and lower toxicity. Moreover, the potential of gossypol to synergize with standard cancer chemotherapies and radiotherapies opens new avenues for its use as a supportive treatment in oncology. The wide distribution of cotton plants, the ease of gossypol extraction, and its proven efficacy against a diverse array of cancer types position it as a highly promising and accessible natural polyphenol molecule. The next step in this journey involves more extensive clinical trials, preferably with larger patient cohorts, to validate its efficacy and safety at a broader scale.

## Conclusion

This paper has assessed gossypol's anticancer properties, highlighting its mechanisms of action, including apoptosis induction, autophagy, angiogenesis inhibition, and potential in immunotherapy. While its transition from a contraceptive to a potential anticancer agent has been notable, the findings underscore the need for cautious optimism. Gossypol’s efficacy, evident in various in vitro and in vivo studies, and its progression through phase I and II clinical trials underscore its potential. However, significant challenges, particularly its narrow therapeutic index and toxicity concerns like irreversible sterility and hypokalemia, are obstacles to its widespread clinical adoption. The exploration of gossypol derivatives offers a promising approach to mitigate these concerns. These derivatives aim to reduce toxicity while maintaining or enhancing anticancer effects. Additionally, the potential synergy of gossypol with standard cancer treatments could broaden its application in oncology. Future research should focus on extensive clinical trials to establish a definitive safety and efficacy profile for gossypol and its derivatives. Investigations into optimized formulations, delivery methods, and combination therapies are essential to fully realize its therapeutic potential. The global availability of cotton plants, as a source of gossypol, further supports its potential as an accessible anticancer agent. In summary, gossypol presents as a compound with significant anticancer potential. However, realizing this potential requires a balanced approach that considers both its promising anticancer properties and the challenges it poses. Continued research is crucial for determining its role in future cancer treatment regimens.

## Data Availability

Not applicable.

## Abbreviations

A(H1N1): Pandemic swine flu
AE: Adverse events
ARE: Antioxidant-responsive element
ATP: Adenosine triphosphate
BALB: Bagg albino (inbred research mouse strain)
Bax: BCL2 associated X
Bcl-2: B-cell lymphoma-2
BH3: Bcl-2 homology domain 3
CCL2: Chemokine (C–C motif) ligand 2
CUL1: Cullin-1
CUL5: Cullin-5
CX43: Connexin43
Cyt-c: Cytochrome complex
DNA: Deoxyribonucleic acid
EGFR: Epidermal growth factor receptor
EGFR-TKIs: EGFR tyrosine kinase inhibitors
ETM: Epithelial-mesenchymal transition
FAK: Focal adhesion kinase
GLPFNs: Gossypol-loaded Pluronic F127 nanoparticles
Gy: Gray (unit of radiation dose)
H3N2: Influenza A virus subtype H3N2
H5N1: Highly pathogenic avian influenza
HIV-1: Human immunodeficiency virus
HNC: Head and neck carcinoma
ICAM: Intercellular adhesion molecule
IL-6: Interleukin 6
IL-8: Interleukin 8
LC3: Light chain 3
MCF: Michigan cancer foundation-7
MCL1: Myeloid leukaemia cell differentiation protein
MDM: Monocyte-derived macrophages
MDM2: Mouse double minute 2
mOS: Median overall survival
mPFS: Median progression-free survival
NCBI: National center for biotechnology information
NCI: National Cancer Institute
NFkB: Nuclear factor kappa B
NOXA: NADPH oxidase activator
NRF2: Nuclear factor erythroid 2–related factor 2
NSCLC: Non-small cell lung cancer
OS: Overall survival
PERK-CHOP: Protein kinase R-like endoplasmic reticulum kinase - C/EBP homologous protein
PEG-b-PCL: Poly(ethylene glycol)-block-poly(ε-caprolactone)
PFS: Progression-free survival
p.o.: Per oral
RBX1-CUL1: RING-box protein-cullin
ROS: Reactive oxygen species
SAE: Serious adverse events
SAG-CUL5: Surface antigen-cullin
SCID: Severe combined immunodeficiency
SOX9: SRY-box transcription factor 9
SRY: Sex determining region Y
TAZ: Transcriptional coactivator with PDZ-binding motif
TGF: Transforming growth factor
TLR4: Toll-like receptor 4
TNF-α: Tumour necrosis factor-alpha
VEGF: Vascular endothelial growth factor
WHO: World Health Organisation
YAP1: Yes-associated protein 1
